# Hospital Abuse and Its Cure

**Published:** 1900-11-17

**Authors:** 


### THE INSTITUTIONAL WORKSHOP.

### HOSPITAL ABUSE AND ITS CURE.

For upwards of thirty years the keenest controversy has raged with varying force around the facts pointing to hospital abuse, its increase and diminution. Few, if any, changes, however, have occurred in the plan upon which hospital relief is granted, and it has become evident that under it nothing effective can be done to reduce the number of hospital patients in this country. The causes which have led to the continuous increase in the number of patients treated at our medical institutions are mainly: (1) growth in (*a*) the population, (*b*) the popularity of the hospitals, (*c*) public confidence in hospital treatment; (2) competition between medical schools and the desire for clinical material; (3) The great increase in the number of special departments attached to general hospitals; (4) the improved hygienic condition and methods of treatment which have caused a remarkable diminution in hospital mortality.

Taking these causes in order, but grouping them as they affect the opinion and actions of (1) the members of the medical profession and (2) the public, respectively, in regard to this question, we may point out that the growth in population is the least important factor in reality because the voluntary support of hospitals has become a recognised institution with all classes, and so in effect the increase in the annual revenue of the hospitals from voluntary sources has been more than sufficient to defray the cost of treating the increase in the number of patients due to population alone. The causes which have chiefly affected the opinion of the medical profession are the competition between the medical schools and medical teachers, leading to a desire to have as wide a field of selection as possible for the purpose of clinical instruction; and the increase in the number and importance of the departments which have grown up in connection with all our large general hospitals, which has probably doubled the number of patients formerly resorting to these institutions for treatment. As to the first, there can be no doubt that a properly administered hospital under reformed methods could secure the most ample supplies of clinical material without permitting one single case of abuse to enter its walls. All who are familiar with the private clinics of the most famous foreign consultants are aware that although the very wealthiest classes are to be found amongst the patients, the medical directors of these clinics are enabled to use every case at their discretion for purposes of clinical instruction. It follows that whatever system of classification of patients was adopted, and however careful the hospital administration might be to exclude from the benefits of free medical relief every one who could pay something up to an adequate sum for that treatment, the whole of the cases, under proper regulations, which had any clinical interest, whatever the circumstances of the patients individually, could be made available for clinical instruction. It is, we know, impossible at present to make the medical profession in England realise that such is the fact; but a fact it is, for when people are really ill, especially where they have intelligence and training, they will readily agree to every condition of treatment rendered necessary for the efficient conduct of a great medical institution.

When we consider next the special departments of the great hospitals, we fail utterly to see why they should be conducted on an entirely free principle whereby everybody is received without question, when the special hospitals dealing with the same class of disease have, for the most part, from the outset received a considerable income from the contributions of their patients who belong to the same classes as the general hospital patients, but are admitted to treatment under a more modern and, if properly safeguarded, a more wholesome and better system of relief. It follows from this that, so far as medical opinion and the medical profession are concerned, every adequate change in the system of administering our hospitals which is required to make hospital abuse impossible can be introduced without any appreciable risk; that a serious diminution in the amount of clinical material, or any interference with the adequate treatment of all classes of disease and the most ample instruction of the students attached to the great medical schools will follow such reforms.

### Why the Public Flock to the Hospitals.

Turning next to the reasons which have induced the people of all classes to attend the hospitals in increasing numbers every year, we find that they mainly arise from increased confidence in hospital treatment, and consequently in hospitals as residences for the sick, due largely to the improved hygienic condition of the buildings, and to increased comforts, improved nursing, and the consequent remarkable diminution in hospital mortality represented by the much larger percentage of recoveries nowadays than formerly. These results are no doubt attributable in great measure to improvement in the methods of treatment, and to greater attention to the many details of each case, which not only diminish suffering, but materially improve the prospects of recovery. Casual readers of the daily press—and nowadays most readers are very casual, if we may judge by the contents of those papers which claim the largest circulation—who frequently see long articles headed "Another Hospital Scandal," would conclude that very few people would care to enter establishments the conduct of which is so severely criticised. The truth, however, is, of course, that every hospital has more cases to handle every year, and that the average of administration of all the great hospitals is so excellent in this country, that the mistakes which occur cannot be much more than one-thousandth part of 1 per cent. of the whole number of patients treated. Hence all classes, if they meet with an accident, are not only content with, but are desirous of being taken at once to, the nearest hospital; and equally, of course, everyone who has not adequate means to defray the cost under most suitable conditions of treating severe illness at home strives to enter a hospital. In these circumstances it must be quite apparent to all who will pause to look at the facts, that there must be hospital abuse, if we define abuse as meaning the taking of free medical relief from a public institution by individuals who can afford to pay a portion or the whole of the cost of their treatment, which they now receive for nothing. Yet, for the reasons given, as we have seen, despite the most active propaganda on the part of interested parties, *i.e.*, members of the public who support hospitals, and who desire that their gifts shall be expended upon the poor as opposed to the well-to-do, and by the great body of medical practitioners who depend for their living upon the classes who can afford to pay the whole or a considerable portion of the cost of their treatment when ill, hospital abuse has continued, has increased, and must still flourish unless there is a radical modification in the present system of administering our hospitals and of distributing free medical relief. In view of the failures of the past if a change for the better is to come, we believe it will come for financial reasons mainly, as we will next proceed to show.

(*To be continued.*)

